# Insulin-Like Growth Factor 1 and Related Compounds in the Treatment of Childhood-Onset Neurodevelopmental Disorders

**DOI:** 10.3389/fnins.2016.00450

**Published:** 2016-09-30

**Authors:** Cyrus Vahdatpour, Adam H. Dyer, Daniela Tropea

**Affiliations:** ^1^School of Medicine, Trinity College DublinDublin, Ireland; ^2^Department of Psychiatry, Trinity College DublinDublin, Ireland

**Keywords:** IGF-1, autism spectrum disorders, Rett Syndrome, Fragile X Syndrome, Phelan-Mcdermid Syndrome

## Abstract

Insulin-Like Growth Factor 1 (IGF-1) is a neurotrophic polypeptide with crucial roles to play in Central Nervous System (CNS) growth, development and maturation. Following interrogation of the neurobiology underlying several neurodevelopmental disorders and Autism Spectrum Disorders (ASD), both recombinant IGF-1 (mecasermin) and related derivatives, such as (1-3)IGF-1, have emerged as potential therapeutic approaches. Clinical pilot studies and early reports have supported the safety/preliminary efficacy of IGF-1 and related compounds in the treatment of Rett Syndrome, with evidence mounting for its use in Phelan McDermid Syndrome and Fragile X Syndrome. In ASD, clinical trials are ongoing. Here, we review the role of IGF-1 in the molecular etiologies of these conditions in addition to the accumulating evidence from early clinical studies highlighting the possibility of IGF-1 and related compounds as potential treatments for these childhood-onset neurodevelopmental disorders.

## Introduction

Insulin-Like Growth Factor 1 (IGF1) is a polypeptide hormone and a member of a superfamily of related insulin like hormones termed Insulin Like Peptides (ILPs) (Fernandez and Torres-Alemán, 2012). IGF-1 is primarily released by hepatocytes in response to Growth Hormone, but is also produced in the Central Nervous System (CNS) where it has pleiotrophic effects on all major CNS cell types (Bach et al., 1991; O'Kusky and Ye, 2012). IGF-1 has crucial roles to play in the development, growth and maturation of the CNS and its synapses, roles which have been reviewed extensively elsewhere (Bach et al., 1991; Popken et al., 2004; Aberg et al., 2006; Llorens-Martín et al., 2009; Corvin et al., 2012; O'Kusky and Ye, 2012; Supeno et al., 2013; Huat et al., 2014; Dyer et al., 2016).

IGF-1 and IGF1 receptor (IGF1R) expression levels have a definite spatio-temporal patterns (Bach et al., 1991; Bartlett et al., 1992; Bondy and Lee, 1993; Zhang et al., 2007). Of note, the abundance of IGF-1R expression over IGF-1 hints at the importance of peripherally produced IGF-1 in mediating the effects of IGF-1 on the CNS (Fernandez and Torres-Alemán, 2012). IGF-1 acts on its glycoprotein receptor (IGF1R), a tyrosine kinase receptor, to activate canonical signaling pathways, including: (i) the PI3K (phosphatidylinositol-3 kinase)—AKT1 (serine-threonine-specific protein kinase)—FOXO (forkhead box protein O) and (ii) the RAS—MAPK (mitogen-activated protein kinases)—ERK (extracellular signal regulated kinases) pathway. Both of these pathways have important roles to play including cell cycle regulation, gene expression, protein synthesis, autophagy, apoptosis and remodeling of the cytoskeleton (Fernandez and Torres-Alemán, 2012; Costales and Kolevzon, 2015).

Once released in the serum, IGF-1 can be cleaved to yield an amino terminal glycine-proline-glutamate (GPE tripeptide) and a truncated IGF-1 form called des-N-(1-3)-IGF-1, lacking the N-terminal GPE tripeptide, which has greatly reduced affinity for IGFBPs and is therefore, is more potent than IGF-1. (1-3)IGF-1 is an active metabolite with neuroprotective effects as well as effects on excitatory synaptic markers such as synapsin 1 and post-synaptic density 95 (PSD-95), recapitulating many of the effects of IGF-1 on synaptic maturation and plasticity (Guan et al., 1999; Corvin et al., 2012). Of note, this effect of (1-3) IGF-1 may be different in neuronal and non-neuronal cell populations. In one report studying the effects of IGF-1 acting on its canonical pathways in neuronal and non-neuronal cells, IGF-1 was found to increase pAkt statining in neurons but not glial cells, whilst (1-3) IGF-1 increased staining in glial cells but not neurons (Corvin et al., 2012). Thus, signaling by IGF-1 and its active tripeptide may demonstrate different effects in different CNS cell populations, adding further to the biological complexity of IGF-1 signaling. One mechanism of action of (1-3)IGF-1 may be to indirectly activate the IGF-1R via an increase in endogenous IGF-1 release (Corvin et al., 2012). Another important aspect of IGF-1 physiology is its binding to IGF Binding Proteins (IGFBP), which regulate its bioavailability, localization and activity (Ocrant et al., 1990; Clemmons, 1998; Hwa et al., 1999; Firth and Baxter, 2002).

Disruption of IGF-1 function has profound phenotypic consequences both in murine models and in humans, underscoring the important role of IGF-1 in CNS development and maturation (Beck et al., 1995; Woods et al., 1996; Netchine et al., 2011). In the present Mini-Review, we address the role of perturbed IGF-1 signaling and the therapeutic potential of IGF-1 and (1-3)IGF-1 in neurodevelopmental disorders such as Rett Syndrome (RTT), Fragile X Syndrome (FXS), Phelan McDermid Syndrome (PMDS) as well as broader Autism Spectrum Disorder (ASD).

## Rett syndrome

Rett Syndrome (RTT) is a pervasive X-Linked neurodevelopmental disorder affecting 1:10,000 female (Percy and Lane, 2004). RTT is characterized by an apparently normal development, followed by a subsequent regression in psychomotor, social and cognitive abilities, deficits in social interaction and a loss of acquired fine motor skills and purposive hand movements. In the CNS, RTT is characterized by microcephaly, neuronal atrophy and leads to cardiorespiratory problems (Julu et al., 2001; Hagberg, 2002). At present, the treatment of RTT represents an unmet therapeutic need.

More than 85% of RTT is caused by a mutation in methyl CpG-binding protein 2 (*MeCP2*), encoding the protein MECP2, which has roles both inside and outside the nucleus (Kaufmann et al., 2005; Chahrour et al., 2008). Atypical cases of RTT may also be caused, in less than 10% cases, by mutations in cyclin-dependent kinase-like 5 (CDK-L5) and in the Forkead Box G1 (FOXG1) in 1% cases (Cahrour and Zoghbi, 2007).

The deletion of the *MeCP2* gene in mouse models recapitulates many of the autonomic, motor and cognitive features of the human RTT phenotype (Banerjee et al., 2012; Castro et al., 2014; Katz et al., 2016), together with reduced connectivity (Armstrong, 2005; Chapleau et al., 2009) and defects in neurotransmitter and receptor expression. *MeCP2* loss in mice results in an alteration in excitatory-inhibitory balance with reduced excitation/increased inhibition in cortical samples/tissue (Dani et al., 2005; Durand et al., 2012).

*MeCP2* deficient brains demonstrate large numbers of modest transcriptional changes, both positive and negative (Katz et al., 2016). One well characterized target of MECP2 function is Brain Derived Neurotrophic Factor (BDNF), an important modulator of CNS growth, which shows synergy with IGF1 in the CNS (Ding et al., 2006). BDNF is down-regulated in murine models of RTT as well as patients with RTT (Chang et al., 2006; Zhou et al., 2006). Although studies in rats have shown that there is clear exchange of BDNF between the brain and the periphery (Pan et al., 1998) and *viceversa* (Poduslo and Curran, 1996), other studies have failed to raise BDNF in the brain to therapeutic levels, suggesting crossing through the blood-brain barrier (BBB) of this neurotrophin to be insufficient for clinical purposes (Pardridge et al., 1994). IGF-1 is a potential alternative, which on crossing the BBB acts on the same pathways as BDNF (such as the PI3K-Akt and MAP-ERK pathways) and appears important for BNDF effects on activity dependent plasticity (Pardridge et al., 1994; Ding et al., 2006).

In *Mecp2* mutant mice, administration of both IGF-1 and (1-3)IGF-1 reverses many of the features of the RTT phenotype (Chen and Russo-Neustadt, 2007; Castro et al., 2014). Castro et al. (2014) demonstrated reduced IGF-1 levels in *MeCP2* mutant mice, with subsequent daily administration of IGF-1 resulting in an improved lifespan, weight and autonomic parameters in the knockout mice. IGF-1 significantly improved abnormalities in activity dependent plasticity in *MeCP2* mutant mice (using a monocular deprivation paradigm). Similar effects are observed with (1-3) IGF-1 administration (Tropea et al., 2009), together with improved spine density, synaptic amplitude and increased excitatory synaptic markers (Tropea et al., 2009). Furthermore, in a mouse model of atypical RTT with mutations in CDLK5, IGF-1 was demonstrated to rescue deficits in dendritic spine instability and expression of PSD-95 adding further support to IGF-1 as a potential treatment in RTT (Della Sala et al., 2016).

One mechanism by which IGF-1 exerts its effects in RTT may be by acting on its canonical signaling pathways (such as PI3K and MAPK pathways as above). Interestingly, a recent study demonstrates that IGF-1 application may actually increase nuclear *MeCP2* transcript and protein (Tropea et al., 2016). Activity dependent plasticity was also shown to modulate MeCP2 expression and this additionally demonstrates the profound effects of IGF-1 on cellular neuroplasticity in the CNS. Restoration of these abnormalities in activity-dependent plasticity may be one important way in which IGF-1 may exert its effects in RTT. Interestingly, MeCP2 may affect IGF-1 levels by regulation of IGFBPs which has been demonstrated for IGFBP3 in both murine models and humans (Itoh et al., 2007).

IGF-1 is already indicated in the pediatric population for severe growth failure and IGF-1 deficiency. In RTT patients, two early studies have been performed demonstrating the tolerability and safety of IGF-1 as a potential treatment (Pini et al., 2012; Khwaja et al., 2014). Khwaja et al. (2014) demonstrated the safety of IGF-1 in 12 patients with *MeCP2* mutations (9 with RTT) with a 4 week multiple ascending dose (40–120 ug/kg bd) followed by a 20 week open label extension, without any serious adverse events or hypoglycamia. A previous clinical study had demonstrated safety in 6 patients with RTT receiving twice daily injections of IGF-1 for a 6 month period (Pini et al., 2012). This was followed by a single case study of one of the patients reporting the safety of repeated treatment with IGF-1 for a second 6 month cycle (Pini et al., 2014).

Preliminary efficacy analysis by Khwaja et al. (2014) demonstrated an improvement in cardiorespiratory parameters, some neurobehavioral parameters and EEG measures of mood and anxiety (reversed frontal alpha band asymmetry). Pini et al. analyzed 10 patients whom had received IGF-1 treatment in a clinical study and compared various parameters to age and disease severity matched controls (Pini et al., 2016). They reported a significant improvement in disease severity as assessed by clinicians as well as two independent and blinded observers using a novel video based scoring system. Whilst this evidence is preliminary, this early data demonstrating efficacy of IGF-1 in RTT is encouraging. The results of ongoing Phase 2 clinical trials using IGF-1 in RTT are eagerly awaited (NCT01777542). Furthermore, an analog of (1-3)IGF-1, NNZ-2566 (administered orally) has demonstrated efficacy in both clinician and caregiver assessments in an industry-led Phase 2 trial on patients with RTT aged 15-45 (NCT01703533), with a Phase 2 trial on younger patients in progress (NCT02715115). Taken together, there is encouraging evidence for the use of IGF-1 and (1-3) IGF-1 in the treatment of RTT (see Tables 1, 2).

**Table 1:** **Previous studies and trials examining IGF-1 as a potential treatment in several childhood-onset neurodevelopmental disorders**.

**References**	**Disorder**	**Treatment**	**Dose**	**Sample SIze**	**Study type**	**Treatment duration**	**Findings**	**Conclusion**	**Company (if applicable)**
Khwaja et al., 2014	RTT	rhIGF-1	40–120 ug/kg bd (MAD) followed by 12 weeks at max. dose	12	Clinical trial	6 months	Improvement in apnoea index neurobehavioural parameters, measures of mood and anxiety Reversal of alpha band desynchronization on EEG	Safety and preliminary efficacy supported	–
Pini et al., 2012	RTT	rhIGF-1	0.05 mg/kg bd first and last week; 0.1 mg/kg bd in between	6	Clinical study	6 months	No adverse events	Safety of IGF-1 supported	–
Pini et al., 2014	RTT	rhIGF-1	0.1 mg/kg bd	Single case study		6 months	No adverse events	Demonstrated safety of repeated doses in a single patient	–
Pini et al., 2016	RTT	rhIGF-1	0.05 mg/kg bd first and last week; 0.1 mg/kg bd in between	10 (incl. Pini et al., 2012)	Clinical study	6 months	Significant improvement in Rett Severity Score (RSS) and International Scoring System (RSS) Significant improvement in social/cognitive testing endurance	Prelimary efficacy of IGF-1 supported	–
NCT01703533	RTT	NNZ-2566	35 mg/kg or 70 mg/kg bd (note: oral administration)	56	Phase II Trial	28 days	No adverse events Significant improvement in Motor-Behavior Assessment Change Index, Clinical Global Impression of Improvement and Caregiver Top 3 Concerns	Safety and preliminary efficacy supported	Neuren Pharmaceuticals Ltd. see disclosure (Neuren Pharmaceuticals Ltd.)^a^
NCT01894958	FXS	NNZ-2566	35 mg/kg or 70 mg/kg bd (note: oral administration)	45	Phase II Trial	56 days	No serious adverse events Significant improvement in group and individual level analysis of specified core measures	Safety and preliminary efficacy supported	Neuren Pharmaceuticals Ltd.^b^
Kolevzon et al., 2014	PMDS	rhIGF-1	0.04 mg/kg bd to a maximum of 0.12 mg/kg bd	9	Phase II Trial	3 months	No serious adverse events Significant improvement on both the Aberrant Behavior Checklist and Repetitive Behavior Scale	Safety and preliminary efficacy supported	Neuren Pharmaceuticals Ltd.

RTT, Rett Syndrome; FXS, Fragile X Syndrome; PMDS, Phelan McDermid Syndrome, ASD, Autism Spectrum Disorder.

a Neuren Pharmaceuticals Ltd. ASX Announcement 7th Dec 2015. Melbourne, Australia. Neuren's trofinetide successful in proof of concept Phase 2 clnical trial in Fragile X Syndrome. http://www.neurenpharma.com/IRM/PDF/1557/TrofinetidesuccessfulinPhase2trialinFragileX Last accessed 28th July 2016.

b Neuren Pharmaceuticals Ltd. Neuren (Neu) ASX Announcement 12th Nov 2014. Melbourne, Australia. Neuren's NZ-2566 successful in demonstrating clinical benefit in Rett syndrome Phase 2 trial. http://www.neurenpharma.com/IRM/PDF/1447/NeurensuccessfulinRettsyndromePhase2trial Last accessed 28th July 2016.

**Table 2:** **Ongoing trials examining IGF-1 as a potential treatment in several childhood-onset neurodevelopmental disorders**.

**References**	**Disorder**	**Treatment**	**Dose**	**Sample size**	**Study type**	**Treatment duration**	**Findings**	**Company (if applicable)**
NCT01777542	RTT	rhIGF-1	Unknown	In progress	Phase II Trial	10 months	In progress	–
NCT02715115	RTT	NNZ-2566	Various	In progress	Phase II Trial	11 weeks (avg.)	In progress	Neuren Pharmaceuticals Ltd.
NCT01970345	ASD	rhIGF-1	0.04 mg/kg bd to a maximum of 0.12 mg/kg bd	In progress	Phase II Trial	12 weeks	In progress	–

RTT, Rett Syndrome; FXS, Fragile X Syndrome; PMDS, Phelan McDermid Syndrome; ASD, Autism Spectrum Disorder.

## Fragile X syndrome

Fragile X Syndrome (FXS) results from a mutation in the *Fmr1* gene, encoding the protein Fragile X Mental Retardation Protein (FMRP1). The disorder is characterized by learning disability, social anxiety and attention deficit disorder, impaired social interactions and seizures as well as an abnormal physical phenotype with macro-orchidism and facial dysmorphism (Hagerman, 1997; Garber et al., 2008). The treatment of FXS represents a largely unmet clinical need.

FMRP, an mRNA binding protein, expressed in neuronal cell bodies and dendrites acts to regulate protein translation at the synapse, with important roles to play in activity-dependent plasticity (e.g., inhibition of translation triggered by mGluR1/5 in response to neuronal stimulation) (Bhakar et al., 2012). FMRP may also have important presynaptic effects on neuronal transmission, mediated via its effects on large conductance Ca^2+^ activated K^+^ channels (Deng et al., 2013).

Several studies have reported disruptions in MAPK/ERK signaling in FXS. Weng et al. (2008) have demonstrated delayed early-phase phosphorylation of ERK in mice deficient in FMRP whilst Curia et al. (2013) demonstrate a resistance to seizures with dephosphorylation of p-ERK in Fmr1 mice (Weng et al., 2008; Curia et al., 2013). In the PI3K pathway, the p110beta catalytic subunit can be regulated by FMRP, with p110beta and PI3K activity elevated in *Fmr1* knockout neurons. This suggests that dysregulation of PI3K signaling may be involved in the synaptic deficits seen in FXS. Indeed, inhibition of PI3K activity may correct dysregulated synaptic protein synthesis, AMPA internalization and spine density defects in knockout neurons (Gross et al., 2010). Thus, alterations in the same canonical pathways stimulated by IGF-1 and other neurotrophins may underlie a large part of the synaptic pathology seen in FXS.

Impressive preclinical evidence comes from a study by Deacon et al. (2015) using an analog of (1-3)IGF-1, NNZ-2566. In *Fmr1* knockout mice, NNZ-2566 demonstrated a significantly reduced brain phospho-ERK and phospho-Akt. Similarly, NNZ-2566 resulted in a significant reduction in spine numbers, which are increased in the *Fmr1* mice in comparison to controls. Improvement in hyperactivity and anxiety, learning and memory deficits was also observed (Deacon et al., 2015). In patients with FXS, a Phase 2 industry-led clinical trial has been completed using NNZ-2566, with clinical improvement in many of the core symptoms of FXS as disclosed by Neuren Pharmaceuticals (NCT01894958). The exact efficacy of IGF-1 related compounds such as NNZ-2566 awaits further clarification, but this early clinical evidence is encouraging.

## Phelan-McDermid syndrome

Phelan-McDermid Syndrome (PMDS) is another monogenic neurodevelopmental disorder and results from deletions in the SHANK3 gene on chromosome 22q13.3. Affected patients demonstrate global developmental delay, severe impairments in speech and intellectual disability (Phelan and McDermid, 2012). The protein product of SHANK3 is a key scaffolding protein present in the post-synaptic density of excitatory synapses, with key roles in activity-dependent plasticity and the functional maintenance of these synapses.

In 3-week old hippocampal neurons treated with siRNA to inhibit SHANK3 synthesis, there was a decreased number and an increased length of dendritic spines (Roussignol et al., 2005). Similarly, application of SHANK3 resulted in the formation of spine-like protrusions containing SHANK3 in aspiny neurons supporting the role of SHANK3 in spine formation and synaptic plasticity. Further, application of SHANK3 increased immunoreactivity for AMPAR subunits in cell body and dendritic spines (but not for GABA subunits), supporting its role in the function and maintenance of excitatory synapses (Roussignol et al., 2005). Using mice deficient in *Shank3*, Bozdagi et al. found reduced amplitude of miniature excitatory post-synaptic currents (mEPSCs) in the hippocampus and impaired Long Term Potentiation (LTP), with only transient spine expansion present (Bozdagi et al., 2010).

In *Shank3* deficient mice, intraperitoneal injection of IGF-1, administered daily for a 2 week period reversed deficits in AMPAR receptors and LTP described above. Similar results were also seen for the active tripeptide (1-3) IGF-1 (Bozdagi et al., 2013). Recently, similar effects for IGF-1 on *Shank3* deficient human neurons has been demonstrated. By using induced pluripotent stem cells (iPSCs) from patients with PMDS and using them to produce functional neurons, Shcheglovitov et al. demonstrated that these neurons had a reduced expression of SHANK3 with accompanying defects in excitatory synaptic transmission as seen in the murine models above (Shcheglovitov et al., 2013). Treatment of the SHANK3 deficient neurons with IGF-1 increased the amplitude and frequency of EPSCs, restored the amplitude of evoked AMPA and NMDAR EPSCs and restored NMDA receptor currents on application of NMDA (Shcheglovitov et al., 2013). IGF-1 also caused a 340% increase in the fraction of puncta expressing PSD-95 in PMDS neurons (Shcheglovitov et al., 2013).

In a double blind, placebo controlled Phase 2 trial reported by Kolevzon et al., the safety and preliminary efficacy of IGF-1 treatment in PMDS were reported on 9 patients with PMDS aged 5–15 (Kolevzon et al., 2014). IGF-1 treatment was associated with significant improvements in social impairment and restrictive behaviors (Aberrant Behavior Checklist and Repetitive Behavior Scale). No serious adverse events occurred with the main side effects including sleep disturbance, hypoglycemia (<50 mg/dL), constipation, increased appetite and mood changes/irritability. This encouraging evidence awaits further confirmation and exploration in further clinical trials in the PMDS population.

## Autism spectrum disorder

Autism Spectrum Disorder is a heterogenous neurodevelopmental disorders characterized by deficits in social interaction and in speech and language with narrowed interests and repetitive behaviors (Wang and Doering, 2015). Autism affects about 3–6 per 1000 of the population, although recent estimates place its prevalence higher at 1 in 68 (CDC, 2012). The most substantial clue to ASD etiology is its substantial heritability (−90%) (Freitag, 2007). ASD demonstrates complex genetics, and whilst it is a genetically heterogenous disorder, evidence repeatedly implicates genes involved in synaptic development, function and activity-dependent plasticity by both common and rare variation (Wang et al., 2009; Hussman et al., 2011; De Rubeis et al., 2014). The potent effects of IGF-1 on synaptic function, maintenance and plasticity make it a potentially attractive target for the treatment of ASDs.

Vanhala et al. reported low levels of IGF-1 in children with autism, however sample sizes were small (*n* = 11) (Vanhala et al., 2006). In 25 young children with a diagnosis of autism, it was subsequently shown that IGF-1 levels were significantly reduced (Riikonen et al., 2001), and in those with a diagnosis of autism, Cerebrospinal Fluid (CSF) IGF-1 was correlated with head size (Mills et al., 2007). On measuring urinary IGF-1 excretion, Anlar et al. (2007) demonstrated that IGF-1 level was significantly lower in autistic children than in age matched controls. In contrast, a larger study has demonstrated significantly higher levels of IGF-1 in children with autism (Marchetto et al., 2016). The exact role of the IGF-1 axis in ASD awaits further clarification.

Interestingly, in a recent report using neurons derived from patients with ASD, Marchetto et al. found a partial rescue of deficits in neuronal networks (neuronal spike number and activity) on application of IGF-1 (Marchetto et al., 2016). At present, clinical trials of IGF-1 in Autism Spectrum Disorder are ongoing, with a phase 2 trial currently recruiting (NCT01970345). The trial aims to pilot the use of IGF-1 as a novel treatment for the core symptoms of ASD. The results of this trial are eagerly awaited. If successful, further trials will be needed in the ASD population to determine the exact efficacy of IGF-1 as a potential treatment, as well as studies to investigate the specific groups of patients with ASD which benefit the most from treatment with recombinant IGF-1.

## Potential molecular mechanisms of IGF1 and derivates in different disorders

There are three potential mechanisms by which IGF-1 may exert its effects in these neurodevelopmental disorders. The first is increased glutamatergic transmission, as seen in various preclinical studies (Tropea et al., 2009; Corvin et al., 2012; Castro et al., 2014; Marchetto et al., 2016). A potential consequence of this includes effects related to increased synaptic potentiation and plasticity (Bozdagi et al., 2013). A second potential action of IGF-1 in these neurodevelopmental disorders includes activation of molecular pathways involved in growth and connectivity (PI3K and MAPK). In RTT models, where these pathways are down-regulated, IGF1 induces an increase in the relative markers of neuronal function (Tropea et al., 2009; Castro et al., 2014), whilst in FXS models, where they are up-regulated, an (1-3)IGF1 analog induces a decrease in the cellular pathways (Deacon et al., 2015). This may be the result of a homeostatic action of IGF-1, in re-establishing basal levels of activity in these canonical signaling pathways. The third potential mechanism relates to effects on transcription. This has been demonstrated in recent studies of IGF-1 on mecp2 transcript (Tropea et al., 2016). More work is needed to clarify the direct and indirect effects of IGF1 and derivates, and their action in different cell types.

## Conclusion

Preliminary evidence is beginning to emerge from well validated murine models and early clinical studies, that treatment with recombinant human IGF-1 (rhIGF-1/mecasermin) and derived compounds may be of benefit in several childhood onset neurodevelopmental disorders. Whilst the evidence base is preliminary, further clinical trials and studies are needed in order to quantify the effects of IGF-1 on patients with these disorders, as well as identifying particular patients which may derive maximum benefit from treatment with IGF-1 and related compounds. The results of ongoing clinical trials are eagerly awaited.

## Author contributions

CV, AD, and DT had substantial roles in the drafting, writing and editing of the review and final manuscript.

### Conflict of interest statement

The authors declare that the research was conducted in the absence of any commercial or financial relationships that could be construed as a potential conflict of interest.
